# Laterally Implanted Pregnancy With Placenta Accreta Spectrum in a Nulliparous Patient: A Diagnostic Challenge

**DOI:** 10.1155/crog/5675416

**Published:** 2026-06-30

**Authors:** Kristen Warncke, Paola Méndez-Ruiz, Rebecca Pedersen, Steven Blaine Holloway, Shivani Patel

**Affiliations:** ^1^ Department of Obstetrics and Gynecology, University of Texas Southwestern Medical Center, Dallas, Texas, USA, utsouthwestern.edu

## Abstract

**Background:**

Laterally implanted pregnancies—including angular, interstitial, and cornual pregnancies—are rare and diagnostically challenging due to overlapping clinical and imaging features. They may mimic a normal intrauterine pregnancy but are associated with significant maternal morbidity. This case describes a prenatally undiagnosed suspected angular pregnancy complicated by placenta accreta spectrum (PAS).

**Case:**

A 39‐year‐old G1P0 woman underwent induction of labor at 37 weeks for fetal growth restriction and gestational hypertension. Postdelivery, retained placenta was noted. Intraoperative evaluation revealed placental tissue in the left cornua concerning for PAS. Placental removal was unsuccessful, and peripartum hysterectomy was performed.

**Conclusion:**

Laterally implanted pregnancies are rare and may be diagnosed only at delivery. Early recognition of abnormal implantation and multidisciplinary management are essential to reduce maternal morbidity.

**Précis**

Angular and laterally implanted pregnancies are difficult to diagnose and manage; thus, early diagnosis and a multidisciplinary team approach are key to minimizing maternal morbidity and mortality.

**Teaching Points**

1. Laterally implanted pregnancies may be difficult to distinguish on routine imaging and should raise concern for abnormal implantation and placenta accreta spectrum (PAS).
2. Early diagnosis and multidisciplinary care improve outcomes.

## 1. Introduction

Laterally implanted pregnancies encompass a spectrum of entities, including angular, cornual, and interstitial pregnancies. The term “eccentric pregnancy” has been used more broadly to describe the implantation within the superolateral aspect of the endometrial cavity. These entities differ in their anatomic location, imaging characteristics, and clinical outcomes.

Angular pregnancies are located within the endometrial cavity medial to the uterotubal junction. Cornual pregnancies, in contrast, occur within the endometrial cavity of a rudimentary uterine horn in the setting of Müllerian anomalies, such as a bicornuate or septate uterus. Interstitial pregnancies refer to implantation within the intramural portion of the fallopian tube, which traverses the muscular wall of the uterus. Distinguishing among these can be challenging, particularly in the absence of early first‐trimester imaging. [1–5]

Angular pregnancy is a rare but potentially life‐threatening condition, and the clinical presentation often mimics that of a normal intrauterine pregnancy or a tubal ectopic pregnancy, making early diagnosis difficult. Unlike ectopic pregnancies, angular pregnancies are intrauterine and can be viable. Patients typically present with abdominal pain, vaginal bleeding, or, in severe cases, hemodynamic instability due to uterine rupture. Delayed diagnosis can lead to significant maternal morbidity and, rarely, mortality [6].

Diagnosis is primarily made using transvaginal ultrasound and occasionally using magnetic resonance imaging (MRI) when findings are inconclusive. Sonographic features described in the literature include the absence of the interstitial line sign, the contiguity of the decidua and endometrium, and an endomyometrial mantle thickness between 5 and 8 mm. However, these criteria are not always consistently applied, and the rarity of an angular pregnancy often results in delayed or missed diagnosis.

Management depends on gestational age, patient stability, and implantation characteristics. Surgical management is typically required, with the extent of resection ranging from cornual and/or uterine wedge resection to hysterectomy in cases of severe bleeding or uterine rupture.

Angular pregnancy is a rare entity, and its true incidence is uncertain given its variability in diagnostic criteria and reporting. We present a case of a prenatally undiagnosed laterally implanted pregnancy, suspected to represent an angular pregnancy in a primiparous patient, complicated by PAS requiring peripartum hysterectomy, and highlight the diagnostic challenges and clinical implications associated with this condition.

## 2. Case

### 2.1. Patient Presentation

A 39‐year‐old G1P0 presented to Labor and Delivery at 37 weeks′ gestation for scheduled induction of labor. Her prenatal course was complicated by fetal growth restriction with normal umbilical artery Dopplers, gestational hypertension, herpes simplex virus infection, cervical dysplasia, and migraines with aura. She had no prior history of pelvic inflammatory disease, uterine infections, uterine instrumentation/surgery (e.g., dilation and curettage, cesarean delivery), connective tissue disease, or known uterine anomalies.

**Figure 1:**
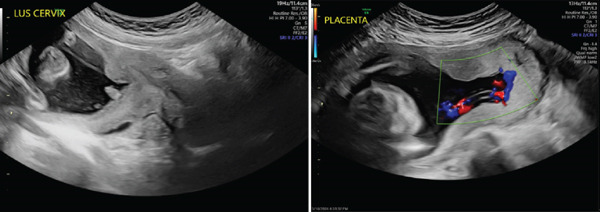
Ultrasound findings of the left lateral placenta with no placenta previa or PAS at 19 weeks′ gestation.

**Figure 2:**
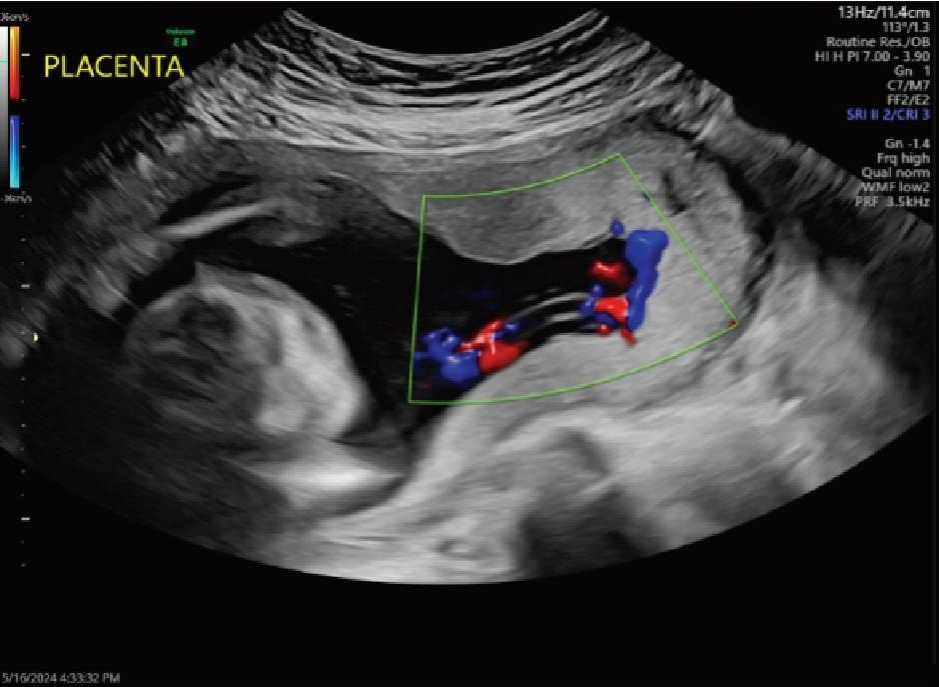
Ultrasound findings of the left lateral placenta without evidence of PAS at 36 weeks′ gestation.

Her first obstetric ultrasound was performed at 7 weeks′ gestation at an outside facility; however, these images and reports were not available for review. She subsequently underwent seven additional ultrasounds for surveillance of fetal growth restriction during pregnancy. At 19 weeks′ gestation, imaging demonstrated a normal‐appearing intrauterine pregnancy with a left lateral placenta, without evidence of placenta previa (Figures 1 and 2). The lateral placental location was not further characterized with respect to the implantation site, though abnormal placentation was not suspected. At 33 weeks′ gestation, fetal growth restriction was diagnosed, and weekly antenatal surveillance was initiated.

### 2.2. Labor and Delivery Course

Given the history of fetal growth restriction and gestational hypertension, induction of labor was initiated at 37 weeks′ gestation with two doses of vaginal misoprostol and artificial rupture of membranes with clear amniotic fluid. The patient progressed to complete cervical dilation and underwent an uncomplicated spontaneous vaginal delivery with a quantitative blood loss of 230 mL. However, the placenta failed to deliver spontaneously after more than 30 min of controlled cord traction. Given concern for retained placenta, the decision was made to proceed to the operating room for dilation and curettage.

### 2.3. Operative Findings

Intraoperatively, both suction and sharp curettage were attempted but were unsuccessful in removing the placenta. Bedside ultrasonography revealed an encapsulated placenta within the left uterine cornu, raising concern for occult PAS. Maternal Fetal Medicine and Gynecologic Oncology services were consulted. The patient remained hemodynamically stable throughout the procedure.

Given failure of conservative management and concern for abnormal placentation, a multidisciplinary decision was made to proceed with exploratory laparotomy. Asymmetric uterine enlargement and focal cornual involvement and ballooning were noted intraoperatively, raising concern for a laterally implanted pregnancy with associated abnormal placentation. Additional efforts at conservative management were unsuccessful, thus necessitating peripartum total abdominal hysterectomy and bilateral salpingectomy. Abnormal and invasive placentation was confirmed after removal of the specimen.

**Figure 3:**
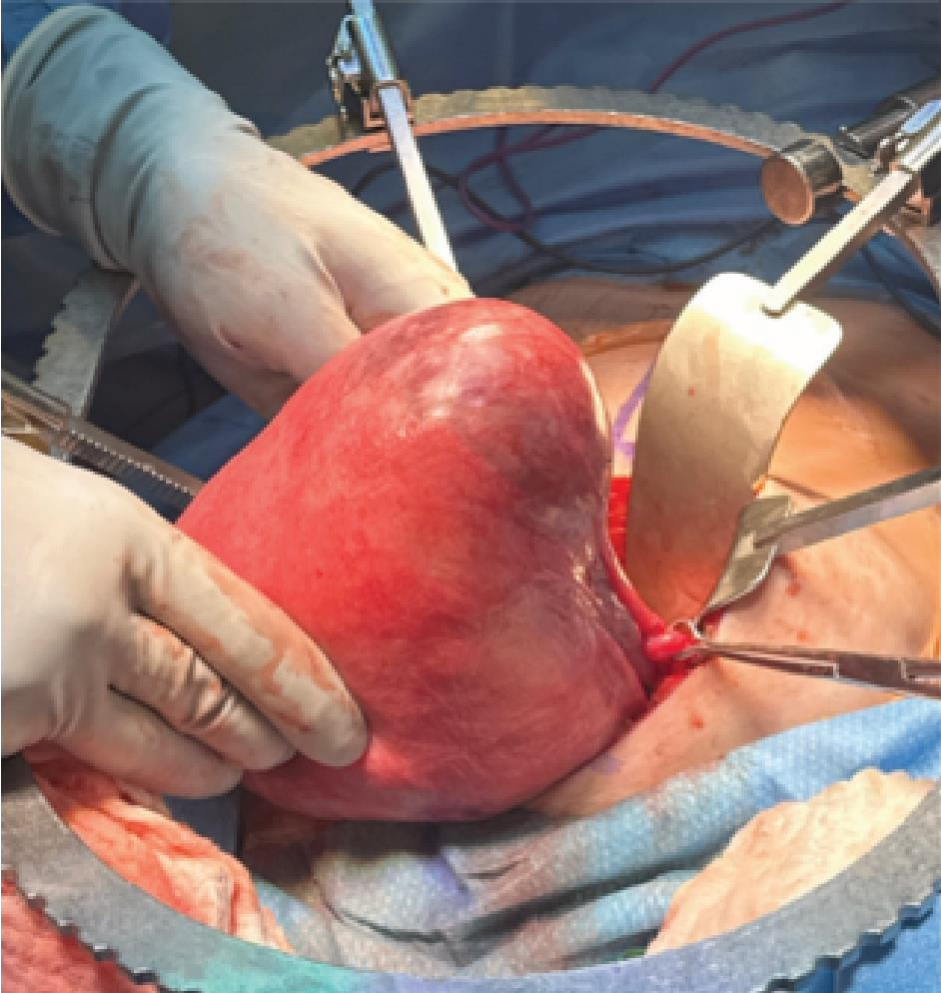
Intraoperative findings of a uterus enlarged to the size of a 20‐week gestation with ballooning of the left uterine cornu.

**Figure 4:**
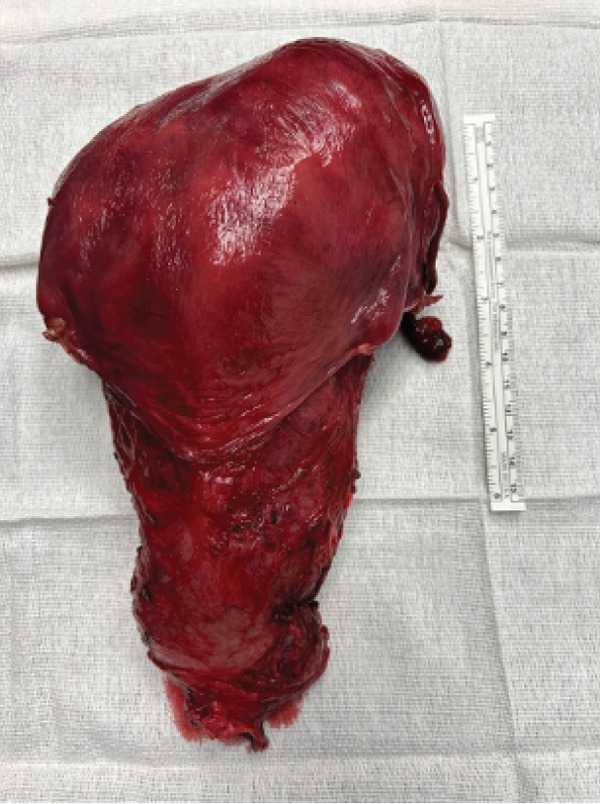
Posthysterectomy enlarged uterus with ballooning of the left uterine cornu.

**Figure 5:**
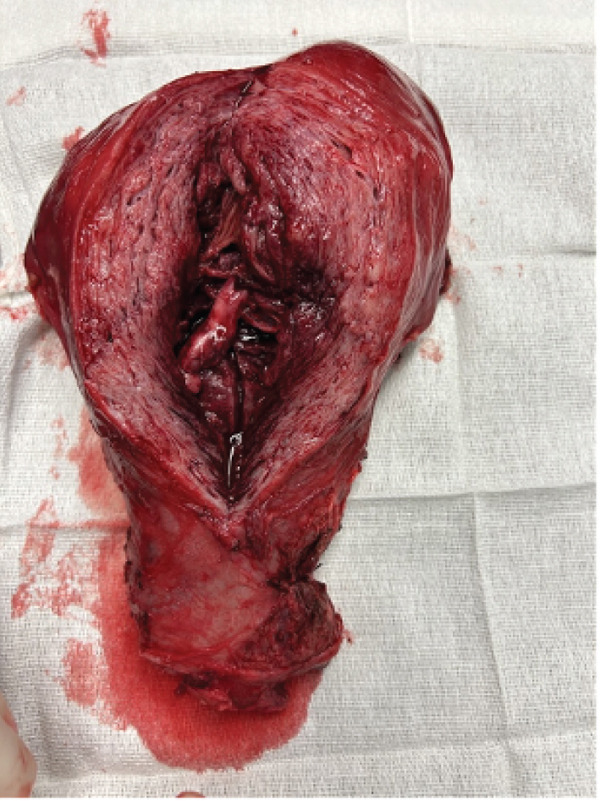
Significant hypervascularity with bridging parasitic vessels.

**Figure 6:**
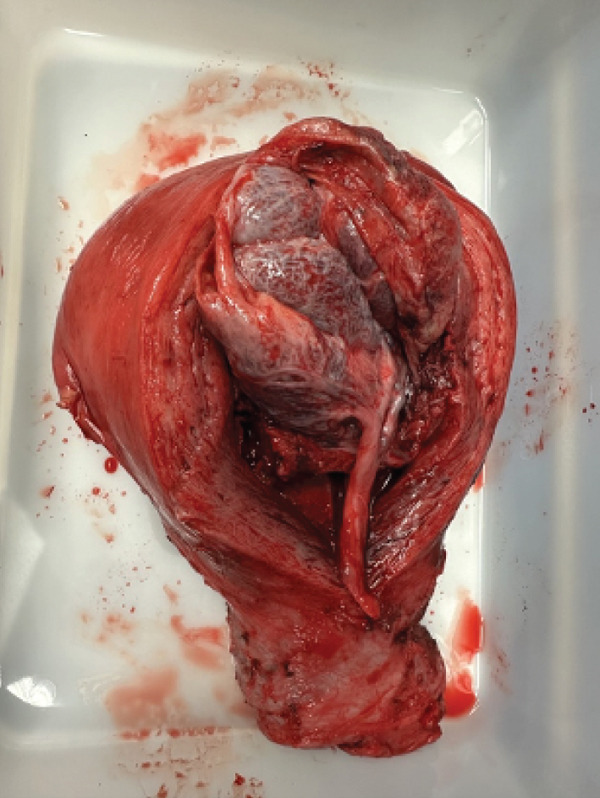
Firm placental tissue within the uterus without serosal invasion.

Notable intraoperative findings included the following:

• A uterus enlarged to the size of a 20‐week gestation with ballooning of the left uterine cornu (Figures 3 and 4).
• Significant hypervascularity with bridging parasitic vessels in the left broad ligament (Figure 5).
• Firm placental tissue within the uterus without serosal invasion (Figure 6).

The cumulative quantitative blood loss from vaginal delivery, dilation and curettage, and hysterectomy was 615 mL. The patient′s hemoglobin remained stable, declining from 12.5 g/dL on admission to 10.7 g/dL intraoperatively and 9.4 g/dL on Postoperative Day 2. The patient did not require a blood transfusion.

### 2.4. Histopathology

Pathologic examination confirmed focal PAS Grade 1, consistent with focal accreta at the left uterine cornu (see the Appendix A section). Findings were localized to the left cornu, corresponding to the area of abnormal placentation identified intraoperatively.

### 2.5. Postpartum Course

The patient′s postoperative course was complicated by preeclampsia with severe features, diagnosed based on persistent severe‐range blood pressure elevations in the postpartum period. She received magnesium sulfate for seizure prophylaxis and met all postpartum and postoperative milestones. She was discharged home in stable condition on Postoperative Day 5.

At her 6‐week follow‐up visit, physical examination demonstrated a well‐healed vaginal cuff. The patient reported new‐onset stress urinary incontinence and was referred to pelvic floor physical therapy. An Edinburgh Postnatal Depression Scale administered 2 weeks postpartum yielded a score of 1, indicating no evidence of postpartum depression.

### 2.6. Neonatal Course

The female infant was born weighing 2150 g (< 1st percentile). Apgar scores were 8 and 9 at 1 and 5 min, respectively. Umbilical arterial blood gas analysis demonstrated a pH of 7.37 with a base excess of −4.

The neonatal course was complicated by respiratory distress requiring supplemental oxygen and treatment for presumed pneumonia. The infant was admitted to the neonatal intensive care unit and discharged in stable condition on Day of Life 11.

## 3. Discussion

Laterally implanted pregnancies represent a diagnostic challenge due to overlapping anatomic definitions and variable imaging findings. In this case, the pregnancy was eccentrically located within the uterine cavity with involvement of the left uterine cornu, raising suspicion for an angular pregnancy. However, a definitive diagnosis could not be established antenatally given the absence of early first‐trimester imaging and the lack of documented sonographic criteria, such as the myometrial mantle thickness.

The differential diagnosis in this case includes angular pregnancy, interstitial pregnancy, and cornual implantation. Angular pregnancies are located medial to the uterotubal junction within the endometrial cavity, whereas interstitial pregnancies occur within the intramural portion of the fallopian tube, and cornual pregnancies are typically associated with Müllerian anomalies [6]. In the absence of early imaging and definitive intraoperative delineation of the implantation site, misclassification among these entities remains possible.

PAS is most associated with prior uterine surgery and placenta previa; however, cases have been reported in patients without identifiable risk factors and in normally situated placentas [7]. Additionally, atypical implantation sites, including fundal and cornual regions, have been described in association with PAS [8]. In this case, the lateralized implantation and cornual involvement raise the possibility that abnormal implantation within the uterine cavity may have contributed to disordered implantation. However, a causal relationship between laterally implanted pregnancies and PAS remains unclear, and current evidence is limited.

This case also highlights the challenges of prenatal diagnosis. Despite multiple ultrasounds performed for fetal growth restriction, no abnormalities in placental implantation were identified. The absence of first‐trimester imaging limited the ability to assess the early implantation site, which is critical for distinguishing laterally implanted pregnancies. Retrospectively, the presence of a persistent lateral placentation, in the setting of unexplained fetal growth restriction in an otherwise low‐risk pregnancy, may represent a subtle clue to abnormal implantation.

Advanced imaging modalities, including targeted transvaginal ultrasound with Doppler and MRI, may improve detection of atypical implantation and abnormal placentation. However, their utility is dependent on clinical suspicion and may not be routinely employed in the absence of suggestive findings.

Earlier recognition of abnormal implantation and placental location may have allowed for anticipatory multidisciplinary planning, potentially reducing the need for emergent surgical intervention. In this case, the patient ultimately required emergent peripartum hysterectomy due to retained placenta and intraoperative findings consistent with PAS. Although the reported patient ultimately experienced a favorable outcome and recovery course, this case serves as a cautionary tale—particularly in care settings where rapid access to high‐complexity, multidisciplinary subspecialty support is not as readily available.

This case underscores several important clinical considerations: (1) laterally located placentas warrant careful evaluation of the implantation site, particularly when identified early in gestation; (2) fetal growth restriction without clear etiology may prompt reassessment of placental location and morphology; and (3) PAS can occur in the absence of traditional risk factors, and a high index of suspicion must persist when faced with an atypical peripartum course. Recognition of these features may facilitate earlier diagnosis and improved peripartum planning, including assembly of a multidisciplinary care team and transfer to a higher level of care facility when appropriate.

## Author Contributions

Kristen Warncke and Paola Méndez‐Ruiz made equal contributions to this work.

## Funding

No funding was received for this manuscript.

## Consent

Patient consent was obtained.

## Conflicts of Interest

The authors declare no conflicts of interest.

## Data Availability

Data sharing is not applicable to this article as no datasets were generated or analyzed during the current study.

## Appendix A: Surgical Pathology for the Uterus, Cervix, Bilateral Fallopian Tubes, and Placenta

Uterus:

- Status postcesarean section
- Focal superficial implantation of chorionic villi on the myometrium without intervening decidua, left lateral/left cornu (see comment)
- Ragged, excavated region, measuring 4.5 × 3.5 cm identified within the left cornu/fundus
- Adenomyosis
- Abundant intramural hemorrhage, edema, and mild acute inflammation

Cervix:

- Acute inflammation, edema, and abundant fresh hemorrhage
- No evidence of dysplasia seen (see comment)

Bilateral Fallopian Tubes:

- Right and left fallopian tubes with congestion and fresh hemorrhage

Placenta:

- 192‐g placenta
- Fragmented maternal surface
- Villous infarct, 0.5 cm
- Distal villous hypoplasia
- Focal increased perivillous fibrin, 1.5 cm

Products of Conception, Dilatation, and Curettage:

- Fragments of chorionic villi, chorionic plate, and myometrium with admixed blood clots and a focal small fragment of stratified squamous epithelium
- The uterus demonstrated occasional chorionic villi in the sections from the left lateral uterus, predominantly superficial/luminal. One of the sections focally shows chorionic villi apparently implanting onto the myometrium without intervening decidua. This is consistent with focal changes of placenta accreta spectrum (PAS), at least focal PAS Grade 1 or focal accreta. History of dilatation and curettage and removal of placental tissue is noted. Due to disruption of the inner uterine wall, deeper abnormal implantation of the placenta cannot be ruled out, and the overall extent of abnormal placental implantation cannot be examined. Multiple sections and deeper levels are examined.
